# High STAP1 expression in DUX4-rearranged cases is not suitable as therapeutic target in pediatric B-cell precursor acute lymphoblastic leukemia

**DOI:** 10.1038/s41598-017-17704-4

**Published:** 2018-01-12

**Authors:** Elisabeth M. P. Steeghs, Marjolein Bakker, Alex Q. Hoogkamer, Judith M. Boer, Quirine J. Hartman, Femke Stalpers, Gabriele Escherich, Valerie de Haas, Hester A. de Groot-Kruseman, Rob Pieters, Monique L. den Boer

**Affiliations:** 1 Department of Pediatric Oncology/Hematology, Erasmus Medical Center – Sophia Children’s Hospital, Rotterdam, The Netherlands; 2 Princess Máxima Center for Pediatric Oncology, Utrecht, The Netherlands; 3 COALL - German Cooperative Study Group for Childhood Acute Lymphoblastic Leukemia, University Medical Centre Eppendorf, Martinistrasse 52, 20246 Hamburg, Germany; 4 DCOG, Dutch Childhood Oncology Group, The Hague, The Netherlands

## Abstract

Approximately 25% of the pediatric B-cell precursor acute lymphoblastic leukemia (BCP-ALL) cases are genetically unclassified. More thorough elucidation of the pathobiology of these genetically unclassified (‘B-other’) cases may identify novel treatment options. We analyzed gene expression profiles of 572 pediatric BCP-ALL cases, representing all major ALL subtypes. High expression of *STAP1*, an adaptor protein downstream of the B-cell receptor (BCR), was identified in *BCR-ABL1*-like and non-*BCR-ABL1*-like B-other cases. Limma analysis revealed an association between high expression of *STAP1* and BCR signaling genes. However, *STAP1* expression and pre-BCR signaling were not causally related: cytoplasmic Igμ levels were not abnormal in cases with high levels of *STAP1* and stimulation of pre-BCR signaling did not induce *STAP1* expression. To elucidate the role of STAP1 in BCP-ALL survival, expression was silenced in two human BCP-ALL cell lines. Knockdown of *STAP1* did not reduce the proliferation rate or viability of these cells, suggesting that *STAP1* is not a likely candidate for precision medicines. Moreover, high expression of *STAP1* was not predictive for an unfavorable prognosis of *BCR-ABL1*-like and non-*BCR-ABL1*-like B-other cases. Remarkably, *DUX4*-rearrangements and intragenic *ERG* deletions, were enriched in cases harboring high expression of *STAP1*.

## Introduction

Acute lymphoblastic leukemia (ALL) is the most common malignancy diagnosed in children. Survival rates have improved during the last decades and nowadays is approaching 90%. This dramatic increase in survival was achieved mostly because of risk-adjusted treatment, therapy intensification, and stem cell transplantations^1^. B-cell precursor ALL (BCP-ALL) can be subdivided in different genetic subtypes, which have different long-term clinical outcome. However, approximately 25% of the BCP-ALL cases lack sentinel genetic aberrations (*KMT2A*-rearrangements, *BCR-ABL1*, *ETV6-RUNX1*, *TCF-PBX1*, high hyperdiploidy) and are classified as ‘B-other’. We and others showed that part of this B-other group has a gene expression profile similar to that of *BCR-ABL1*-positive ALL cases, and is associated with an unfavorable clinical outcome^2,3^. Although these patients are known to lack the *BCR-ABL1* fusion gene, the underlying pathobiology of this subtype remains poorly understood. Cases are enriched for copy number alterations (CNAs) in genes involved in B-cell development, intrachromosomal amplification of chromosome 21, dicentric chromosome (9;20), and a subgroup of cases harbors kinase activating lesions^2–7^. Likewise, the remaining non-*BCR-ABL1*-like B-other cases are a very heterogeneous group. Only very recently, chromosomal translocations involving *DUX4*, *ZNF384*, and *MEF2D* were identified in these non-*BCR-ABL1*-like B-other cases^8–10^. Although kinase activating lesions offer a potentive attractive target for precision medicine, for the remaining group of *BCR-ABL1*-like and non-*BCR-ABL1*-like B-other cases, no druggable targets have yet been identified.

BCP-ALL cells are immature B-cells in which differentiation is arrested at early immature stages. The precursor B-cell receptor (pre-BCR), an immature immunoglubulin (Igµ) heavy chain with ‘surrogate’ light-chain components, is involved in the expansion and maturation of pre-B cells. For a short period of time, in-frame rearranged V_H_DJ_H_ gene segments are expressed to pass the pre-BCR checkpoint. In absence of this expression, pre-B cells are eliminated by programmed cell death^11,12^. Malignant pre-B cells can evade this pre-B cell checkpoint via activation of alternative pathways^12^. In mature B-cells PI3K-AKT signaling can rescue BCR deficient cells^13^. Signaling of pre-BCR and mature BCR are largely similar^14^. Targeting BCR signaling is an attractive treatment strategy in mature B-cell malignancies and is also being explored in BCP-ALL^15–20^.

Advanced understanding of the pathobiology of genetically unclassified BCP-ALL cases may identify novel treatment options. To identify dysregulated genes in these cases, we analyzed gene expression profiles in leukemia cells of cohort of BCP-ALL patients at initial diagnosis^21^. High expression levels of the adapter protein *signal transducing adaptor family member 1* (*STAP1)* were identified in a subgroup of *BCR-ABL1*-like and non-*BCR-ABL1*-like B-other patients. STAP1 is a relatively unknown protein, consisting out of Peckstrin homology (PH) domains and unique Src homology 2 (SH2) domains, suggesting that STAP1 recruits signaling proteins to receptor tyrosine kinases^22,23^. STAP1 is reported to be a docking protein downstream of Tec protein tyrosine kinase (TEC), which is involved in BCR signaling^24,25^. In addition, reports show enriched expression in hematopoietic stem cells and a potential role of STAP1 in microglia activation has been suggested^26,27^. The current study aimed to establish the therapeutic potential of inhibiting STAP1 in the subset of BCP-ALL cases that express high levels of *STAP1*.

## Results

### Discriminative expression profile of *STAP1* in *BCR-ABL1*-like and B-other cases

**Figure 1:**
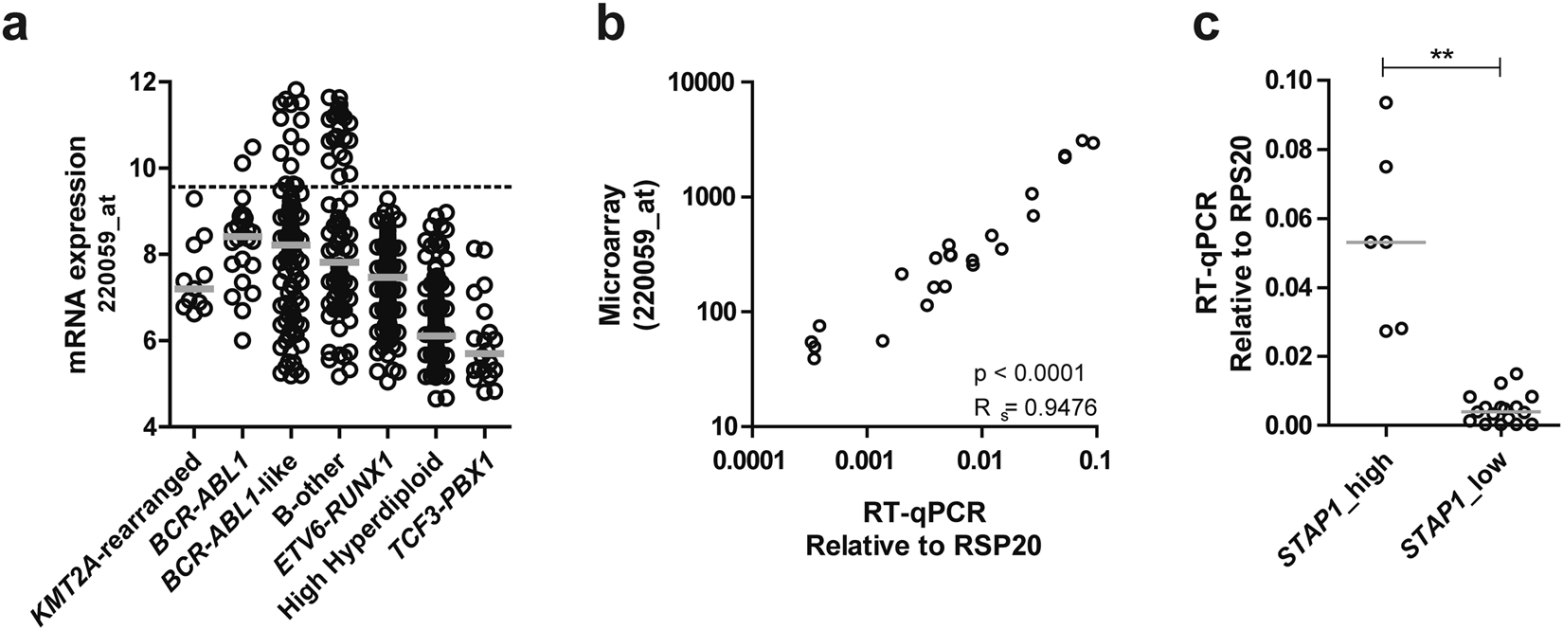
Discriminative expression of *STAP1* in *BCR-ABL1-*like and B-other cases. (**a**) ^2^log expression levels of Affymetrix probeset 220059_at in 572 BCP-ALL cases. The dotted line represents the 80^th^ percentile of *BCR-ABL1*-like and B-other cases. Grey lines represent the median expression values of subtype groups. (**b**) Microarray expression levels were validated using RT-qPCR, as shown by a high spearman correlation coefficient. (**c**) Expression values of *STAP1* in 6 *STAP1*-high and 17 *STAP1*-low cases detected by RT-qPCR. Independent samples T-test. The grey lines represent the median of the *STAP1*-high and the *STAP1*–low group. Mann-Whitney U test. **p < 0.01.

To identify differentially expressed genes in *BCR-ABL1*-like and non-*BCR-ABL1*-like B-other cases, microarrays were performed of a representative pediatric BCP-ALL cohort of 572 cases at initial diagnosis^21^. Limma analyses revealed *STAP1* to be the probeset with the highest fold-change in *BCR-ABL1-*like and non-*BCR-ABL1*-like B-other patients compared to remaining BCP-ALL cases (fold change = 2.88, adjusted p-value < 0.0001; Supplementary Table S1). Strikingly, this elevated expression was characteristic for a subset of patients reflecting about 20% of *BCR-ABL1*-like and non-*BCR-ABL1*-like B-other cases (Fig. 1a, henceforth: *STAP1*-high cases). The remaining *BCR-ABL1*-like and non-*BCR-ABL1*-like B-other cases (henceforth: *STAP1*-low) had *STAP1* expression levels comparable to those observed in other ALL subtypes and mononuclear bone marrow cells of healthy controls (Supplementary Fig. S1). Microarray results were validated using RT-qPCR (Fig. 1b, Supplementary Fig. S1). Subsequently, we analyzed which genes were associated with high expression levels of *STAP1*. Limma analyses revealed 8894 probesets to be differentially expressed between *STAP1*-high and *STAP1*-low cases (adjusted p-value < 0.05; Supplementary Table S2). Ingenuity pathway analysis was performed to identify pathways associated with these differentially expressed genes. EIF2 signaling, mTOR signaling, regulation of eIF4 and p70S6K signaling, B cell receptor signaling (BCR), and AMPK signaling included the top five differentially regulated canonical pathways (Supplementary Fig. S1). Identification of the BCR signaling pathway was a striking observation, as STAP1 is reported to be a docking protein downstream of TEC in this pathway^24,25^. Since the signaling pathways activated by pre-BCR and mature BCR are highly overlapping^14^, we hypothesized a similar function for *STAP1* in the downstream signaling cascade of the pre-BCR. Taken together, the association between *STAP1* and the BCR signaling cascade may suggest an activated pre-BCR signaling pathway in a subset of *BCR-ABL1*-like and non-*BCR-ABL1*-like B-other cases. This hypothesis was further explored since it may offer a targeted treatment strategy for these cases^17–20^.

### High levels of *STAP1* and pre-BCR signaling are not causally connected

**Figure 2:**
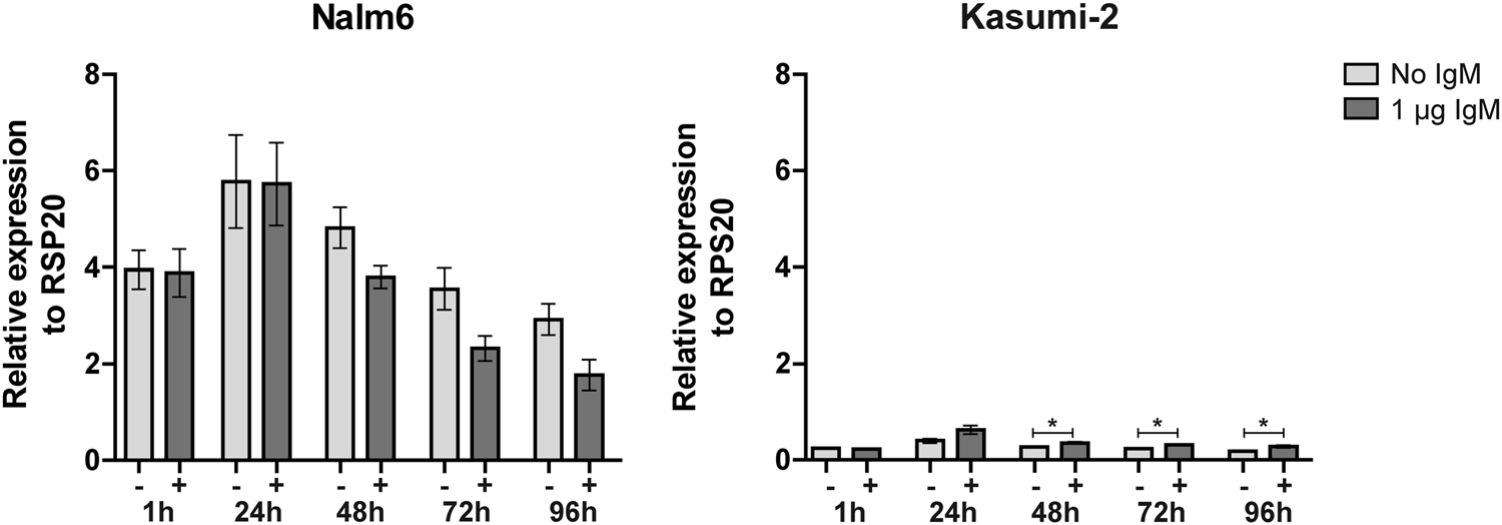
Pre-BCR signaling and *STAP1* expression. The BCP-ALL cell lines Nalm6 and Kasumi-2 were stimulated with 1 µg anti-IgM for 1, 24, 48, 72 or 96 hours. Expression levels of *STAP1* were examined using RT-qPCR. Linear values normalized to RPS20 expression are shown. Mean ± SEM of three independent experiment. Independent samples T-test. * p < 0.05.

We studied whether high levels of *STAP1* associated with high expression of the pre-BCR complex. To determine in-frame rearrangements of immunoglobulin heavy chain V_H_DJ_H_ gene segments (indicative for a functional pre-BCR), cytoplasmic Igµ (CyIgµ) levels were measured in 142 *BCR-ABL1-*like and non-*BCR-ABL1*-like B-other cases. 14 of the 32 (44%) *STAP1*-high cases showed positivity (>30% CyIgµ^+^ cells) compared with 31 of the 110 (28%) *STAP1*-low cases (Fisher exact test. p = 0.13). This result suggests that *STAP1*-high and *STAP1*-low cases do not differ in the number of pre-BCR positive cells. In addition, we examined whether *STAP1* expression itself may be induced by pre-BCR signaling. Therefore, the BCP-ALL cell lines Nalm6 and Kasumi-2 (high and low *STAP1* expression, respectively) were stimulated for 4 days with 1 µg anti-IgM antibody. To confirm activation of pre-BCR signaling by the anti-IgM antibody, phosphorylation levels of AKT^Ser473^ were analyzed (Supplementary Fig. S2). Stimulation of the pre-BCR did not increase *STAP1* expression levels in the *STAP1*-high cell line Nalm6. In the *STAP1*-low cell line Kasumi-2 only a slight increase was detected in time (~1.3–1.6 fold; Fig. 2). Taken together, these results suggest that high levels of *STAP1* and pre-BCR signaling are not causally connected.

### Silencing of *STAP1* does not affect cell viability and drug sensitivity

**Figure 3:**
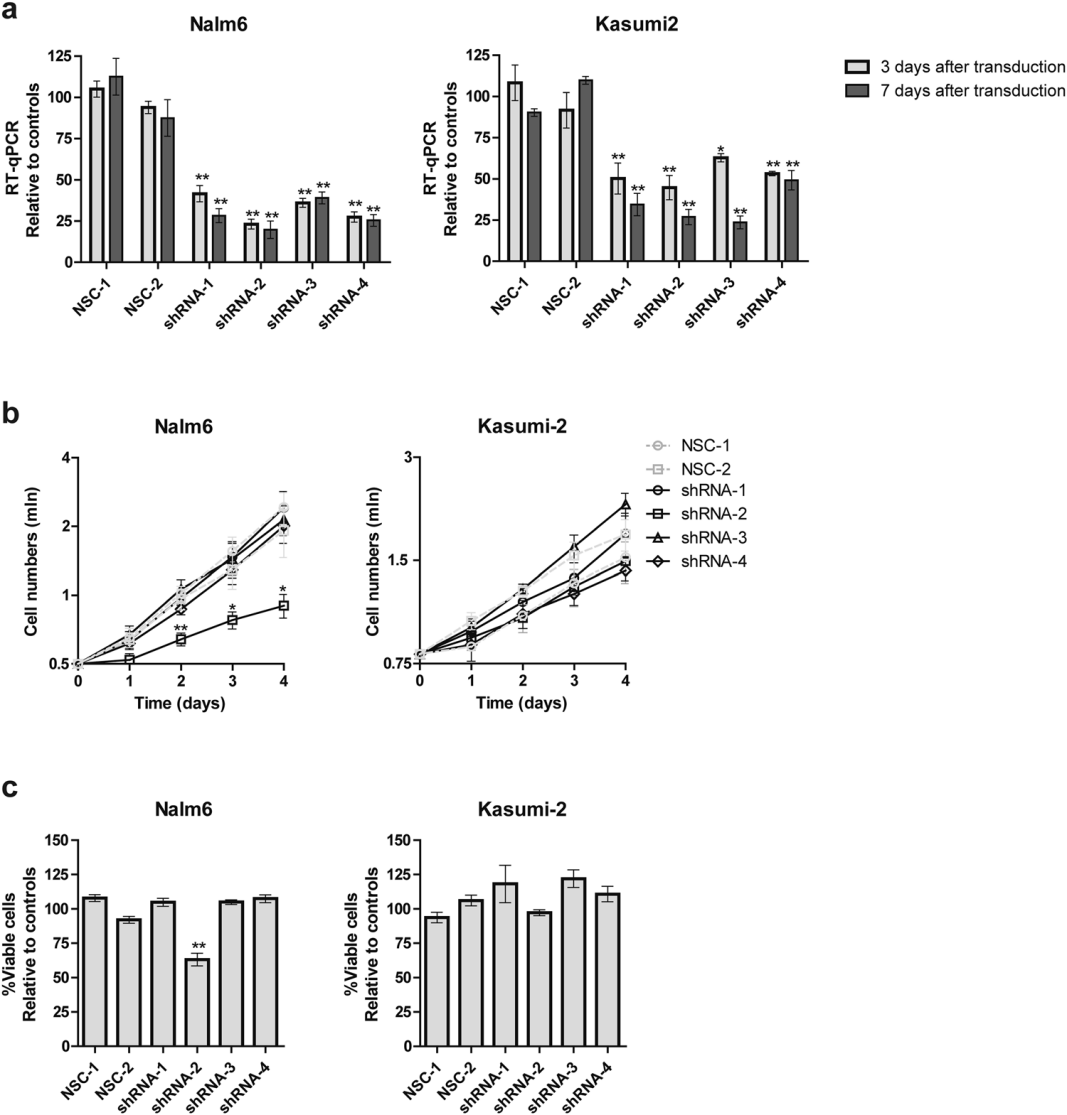
*STAP1* knockdown does not affect leukemic cells survival. Nalm6 and Kasumi-2 cells were transduced via spin-infection with shRNAs targeting *STAP1* or scrambled control vectors. Values represent mean ± SEM of four independent experiments. Independent sample T-test. **p < 0.01; *p < 0.05. (**a**) Knockdown efficacy was determined three and seven days after transduction using RT-qPCR. *STAP1* expression relative to RPS20 was calculated. Relative expression values towards the two scrambled controls are depicted. (**b**) Proliferation of Nalm6 and Kasumi-2 cells was measured for four days. At day 0 (3 days after transduction) cells were plated at equivalent concentrations. The next four days cell concentrations were detected using the MACSQuant and PI staining. Cell numbers (x10^6^) are shown on the y-axis. (**c**) Seven days after transduction, viability of the cells was determined using AnnexinV and PI staining. Viability relative to scrambled control samples is shown.

To elucidate the role of STAP1 in BCP-ALL survival, *STAP1* expression was silenced by four different shRNAs in the BCP-ALL cell lines Nalm6 and Kasumi-2. The knockdown efficiency at mRNA level ranged from 50–80% for both cell lines and was confirmed on protein level (Fig. 3a, Supplementary Fig. S3,4). Three out of four shRNA constructs did not alter the proliferation rate and viability of Nalm6 cells, despite effective knockdown. Only shRNA-2 reduced those parameters in Nalm6 cells, but not in Kasumi-2 cells (Fig. 3b). Seven days after transduction, the majority of leukemic cells remained alive in both cell lines and in all knockdown conditions (Fig. 3c). These data suggest that STAP1 is not essential for the survival of BCP-ALL cells.

**Figure 4:**
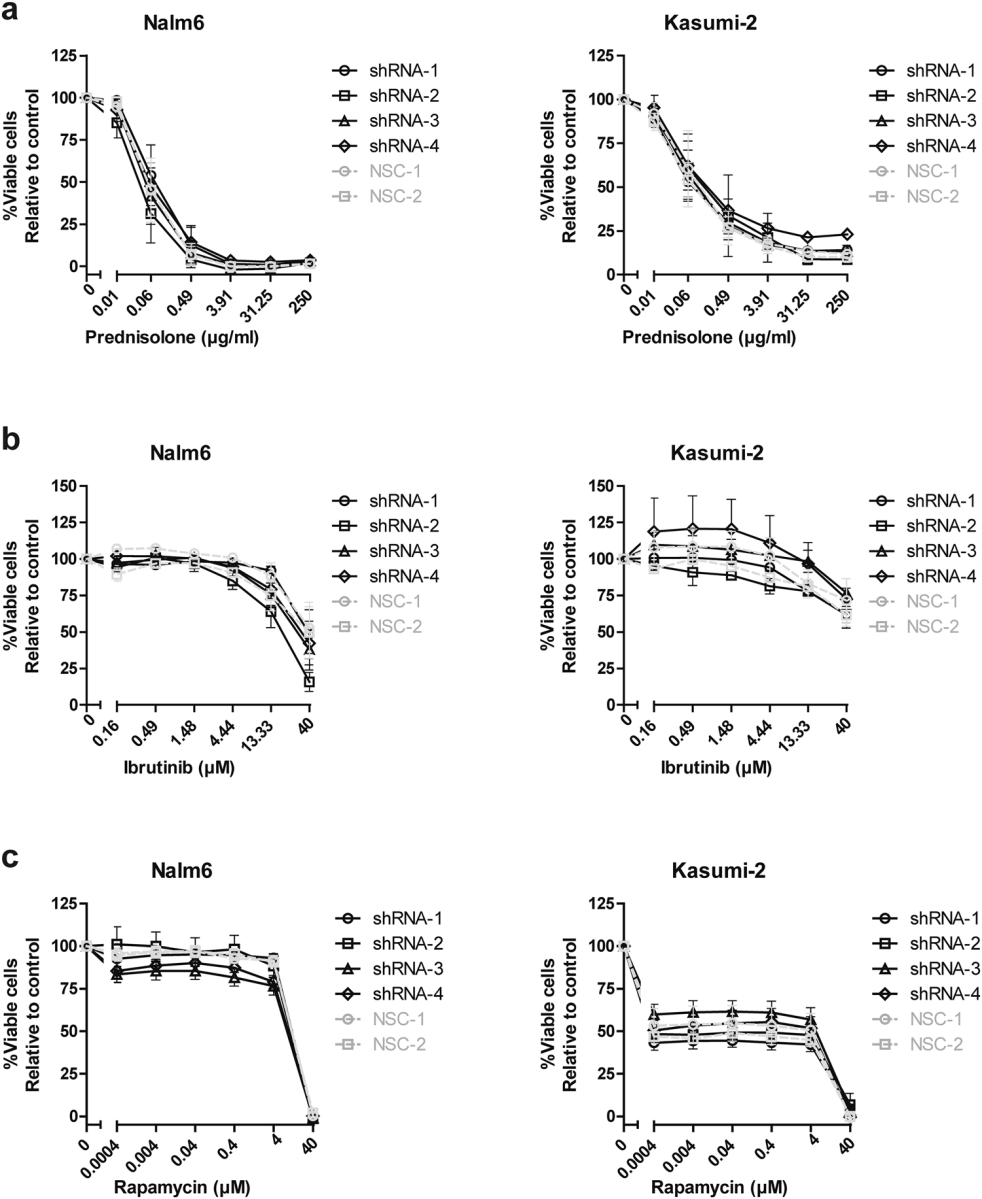
*STAP1* knockdown does not sensitize cells towards prednisolone, ibrutinib or rapamycin. (**a–c**) The efficacy of prednisolone (**a**), ibrutinib (**b**), and rapamycin (**c**) was tested in Nalm6 and Kasumi-2 cells, in which *STAP1* was silenced. Three days after transduction, Nalm6 and Kasumi-2 cells were exposed to a concentration range of the indicated compounds. After four days, viability was quantified using an MTS assay. Values represent mean ± SEM of four independent experiments of Nalm6 and three independent experiments of Kasumi-2.

**Figure 5:**
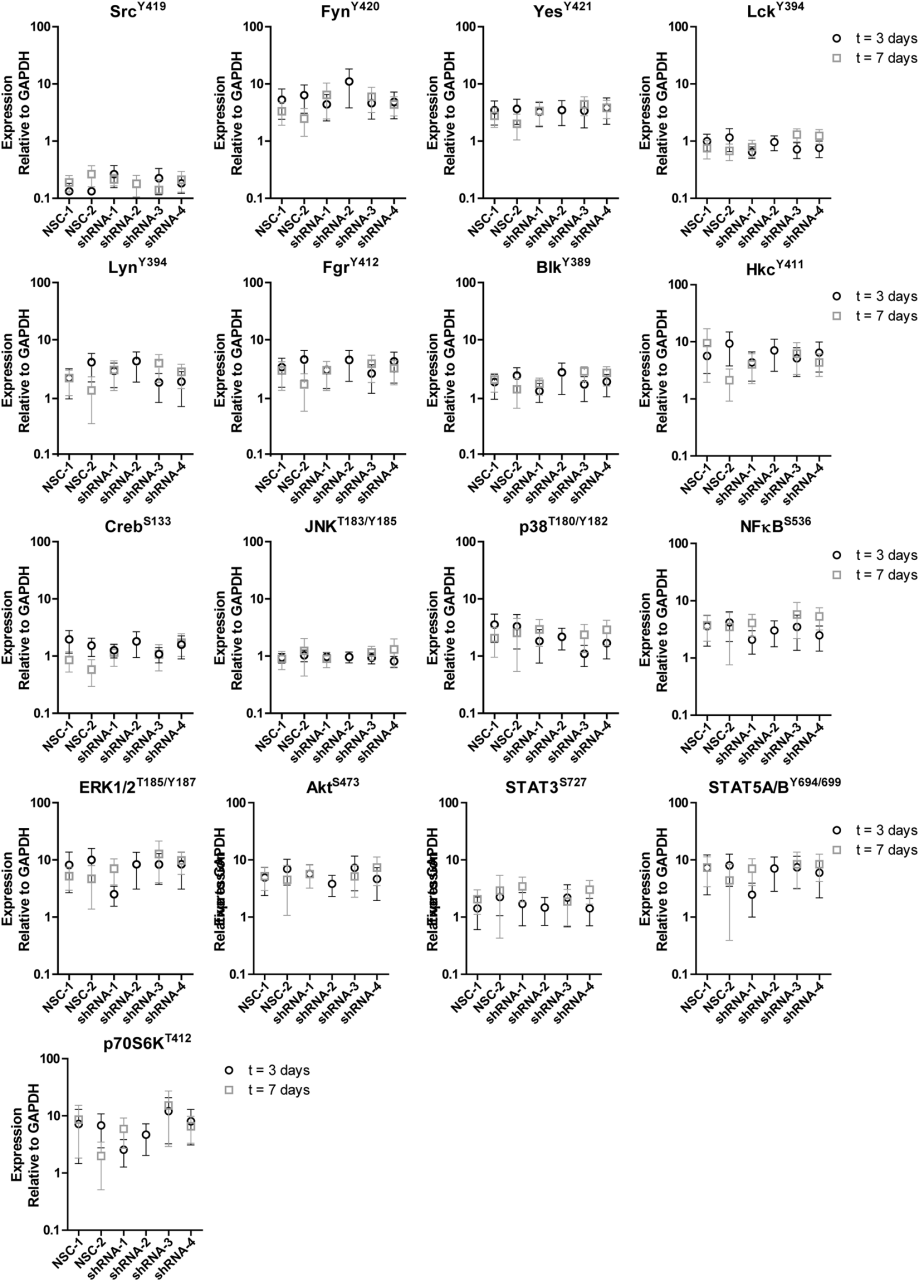
*STAP1* knockdown does not affect common signaling pathways. Expression of 17 proteins was determined using a fluorescent bead-based immunoassay in Nalm6 cells after knockdown of *STAP1*. Fluorescent intensity values relative to GAPDH are depicted. Expression was measured three and seven days after transduction. Infection and selection for stably transduced cells required three days. Subsequently, proliferation, viability and MTS assays were initiated, which took four days. mRNA and protein expression levels were examined after selection (day 3) and at the end of these functional experiments (day 7). At day 7, not enough material was available of Nalm6 cells, which were transduced with shRNA-2. Values represent mean ± SEM of four independent experiments at three days after transduction and three independent experiments at seven days after transduction.

Next, we studied whether silencing of *STAP1* affected sensitivity for inhibitors of pre-BCR (ibrutinib) and mTOR signaling (rapamycin), and the ALL spearhead drug prednisolone. Silencing of *STAP1* did not alter the sensitivity to these compounds in the Nalm6 or Kasumi-2 cell lines (Fig. 4). To elucidate in which other signaling pathway STAP1 may be involved, the phosphorylation status of 17 proteins covering multi-signaling pathways was examined (Fig. 5). Silencing *STAP1* did not alter the phosphorylated levels/ activation of Src-family, PI3K, Ras, Stat, JNK, p38 and NFκB kinase signaling members, nor of the reported STAP1 target in Ramos cells, i.e. phosphorylated level of CREB^Ser133^^25^. Taken together, *STAP1* inhibition did not affect the survival and proliferation of the BCP-ALL cell lines Nalm6 and Kasumi-2 or the phosphorylated levels of downstream signaling molecules, nor did knockdown result into sensitization for prednisolone or signaling inhibitors (ibrutinib and rapamycin).

### Aberrations in B-cell development genes and clinical characteristics

**Figure 6:**
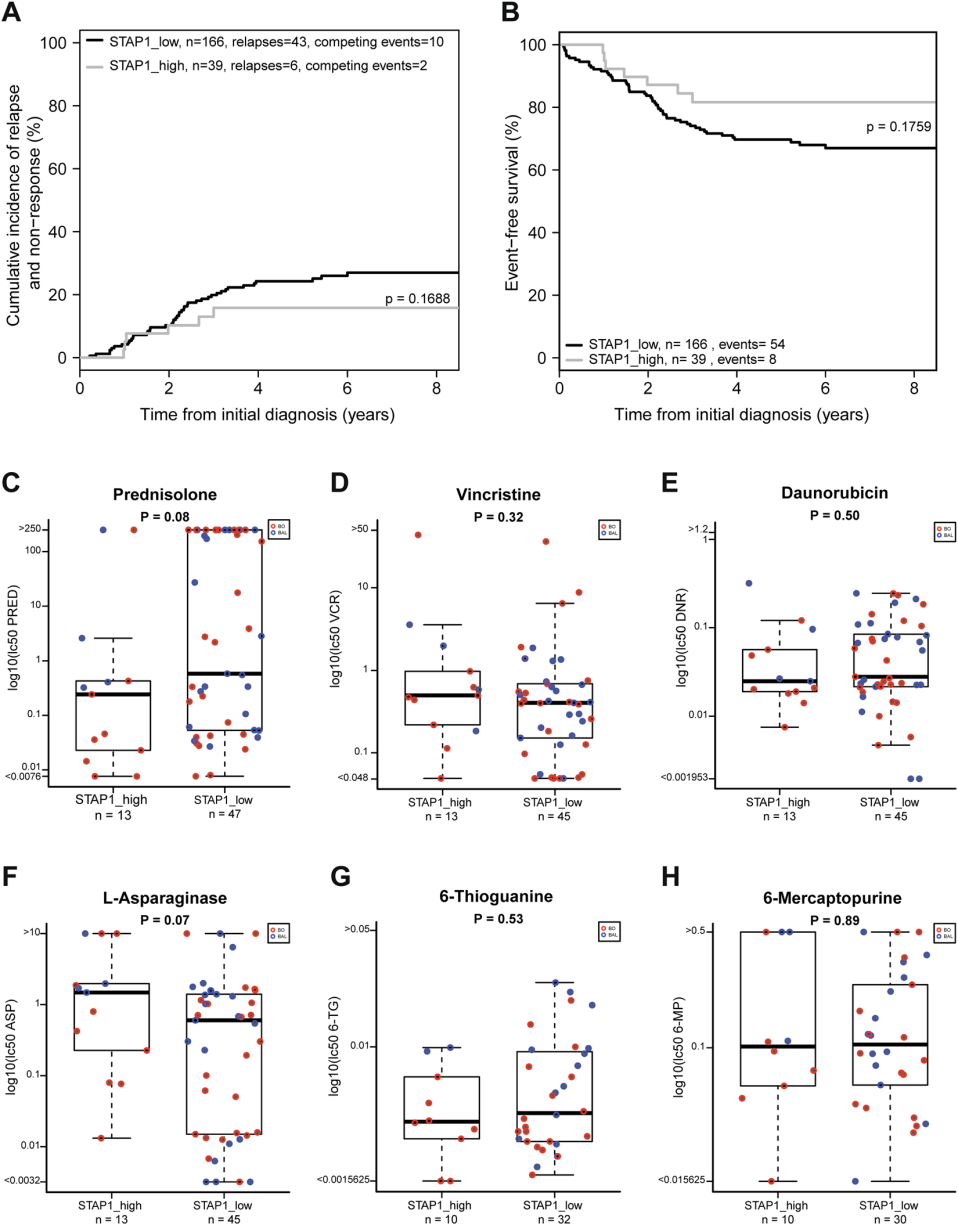
*Ex vivo* drug response and outcome is not affected by *STAP1* overexpression. (**a**,**b**) The association between high expression levels of *STAP1*, and cumulative incidence of relapse (CIR) and event-free-survival (EFS) was examined. Patients were stratified according to treatment protocol (i.e. COALL-97 or -03, or the DCOG protocols ALL8, ALL9, or ALL10). CIR was estimated using a competing risk model with relapse and non-response as event and death as competing event. The Gray’s test was applied. Relapse, non-response, secondary malignancies and death were considered as events for EFS. EFS rates were determined using Cox regression, and compared using the Wald test. (**c–h**) Leukemic cells were exposed for four days to an increasing concentration range of prednisolone (µg/ml), vincristine (µg/ml), L-asparaginase (IU/ml), daunorubicin (µg/ml), 6-mercaptopurine (µg/ml), and 6-thioguanine (µg/ml). Cell survival was measured using an MTT assay. To compare LC50-values, the Mann-Whitney U test was applied. BO = non-*BCR-ABL1*-like B-other BCP-ALL cases. BAL = *BCR-ABL1*-like BCP-ALL cases.’

Genes involved in B-cell development are frequently altered in *BCR-ABL1*-like patients^2,3^. Therefore, frequencies of lesions in genes involved in lymphoid differentiation, proliferation, cell cycle and transcription were studied in *STAP1*-high and *STAP1*-low *BCR-ABL1*-like and non-*BCR-ABL1*-like B-other cases. Copy number alterations affecting *EBF1*, *PAR1*, and *ETV6* were virtually absent in *STAP1*-high cases compared to a frequency ranging from 10.9–22.4% in *STAP1*-low cases (Fisher exact test, p < 0.05; Table 1). More strikingly, high expression of *STAP1* was associated with intragenic deletion of the ETS transcription factor *ERG:* 27.6% (8/29) in *STAP1*-high cases compared to 0.8% (1/133) in *STAP1*-low cases (p < 0.0001, odds ratio = 47.7; Table 1). Deletions of *ERG* are associated with a favorable prognosis in genetically unclassified high-risk pediatric BCP-ALL^28–30^. The five-year CIR and EFS of *STAP1*-high and *STAP1*-low *BCR-ABL1*-like and non-*BCR-ABL1*-like B-other cases did not significantly differ in our cohort, although *STAP1*-high cases showed a trend for a more favorable outcome (Fig. 6a,b). *STAP1*-high cases had a higher median age (Mann-Whitney U test; p = 0.013; 9 years, range 2–16) than *STAP1*-low cases (6 years, range 1–18). Remaining clinical characteristics, i.e. white blood cell count, *in vivo* prednisolone response at day 8, gender, and MRD levels did not differ (Supplementary Table S3). In addition, *ex vivo* cytotoxicity of prednisolone, vincristine, daunorubicin, l-asparaginase, 6-thioguanine, 6-mercaptopurine was measured in primary leukemic cells. No difference in cytotoxicity of these compounds was observed in *STAP1*-high and *STAP1*-low *BCR-ABL1*-like and non-*BCR-ABL1*-like B-other cells (Fig. 6c–h).

**Table 1:** Copy Number Alterations in B-cell development genes.

	STAP1 high cases		STAP1 low cases		P-value	Odds Ratio	95% Confidence Interval	
	Number	Percentage	Number	Percentage				
*IKZF1* deletion	8/36	22.2	58/156	37.2	0.120			
*EBF1* deletion	**0/36**	**0**.**0**	**17/156**	**10**.**9**	**0**.**046**	**0**.**00**	**0**.**00**	**1**.**00**
*PAX5* aberration	10/36	27.8	69/156	44.2	0.091	0.49	0.20	1.13
*CDKN2A/B* deletion	15/36	41.7	77/156	49.4	0.460			
*RB1* deletion	1/36	2.8	14/156	9.0	0.310			
*BTG1* deletion	1/36	2.8	10/156	6.4	0.690			
*ETV6* deletion	**1/36**	**2**.**8**	**35/156**	**22**.**4**	**0**.**004**	**0**.**10**	**0**.**00**	**0**.**64**
*PAR1* deletion	**0/36**	**0**.**0**	**19/156**	**12**.**2**	**0**.**027**	**0**.**00**	**0**.**00**	**0**.**87**
ERG deletion	**8/29**	**27**.**6**	**1/131**	**0**.**8**	**3**.**80E-06**	**47**.**68**	**5**.**91**	**2183**.**65**

Fisher’s Exact test p-values are shown. Values shown in bold if p < 0.05;

Odds ratios are only given if p-values in Fisher’s Exact test were below 0.1.

**Figure 7:**
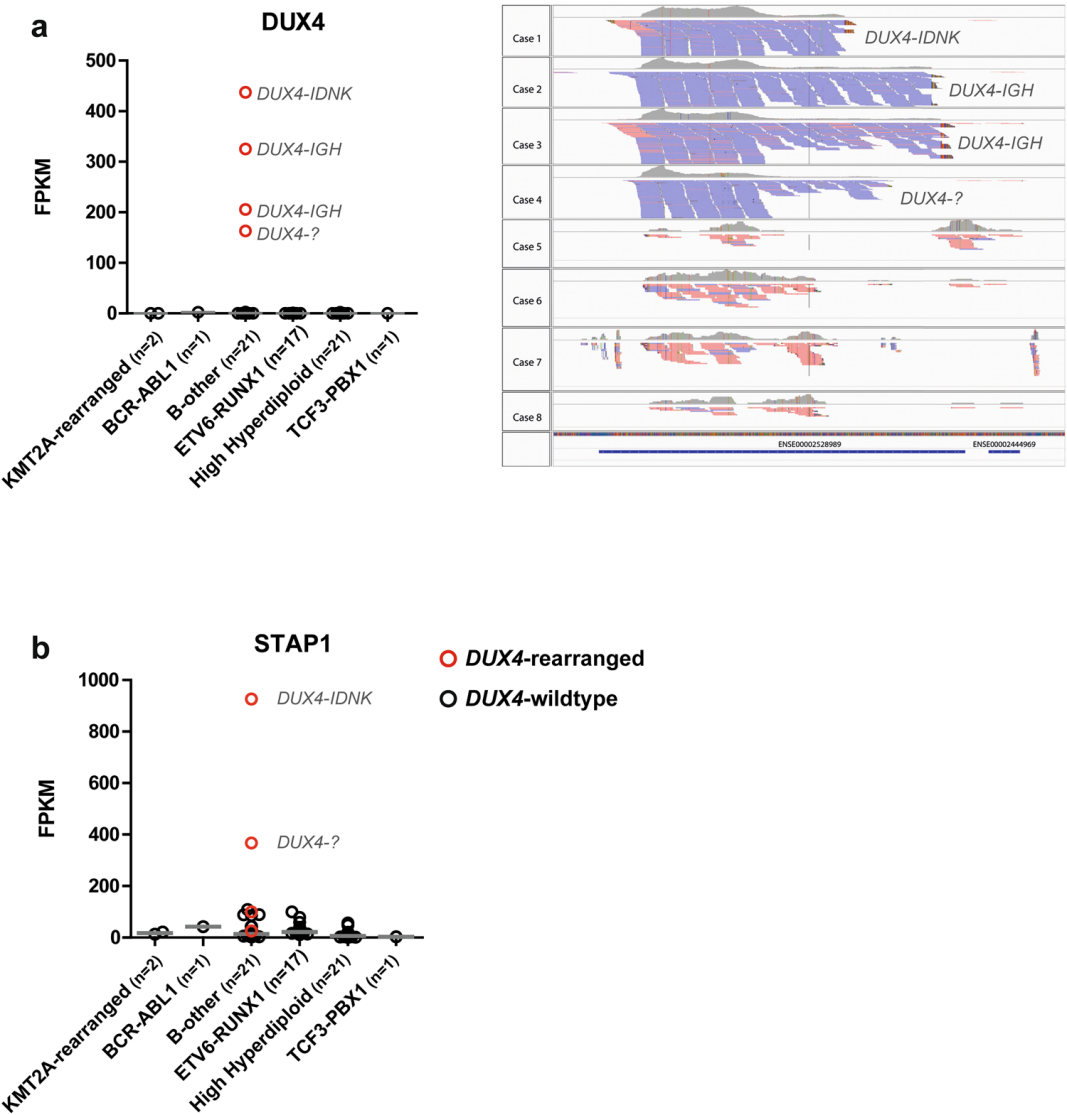
STAP1 expression and *DUX4*-rearrangements. RNAseq analyses were used to determine *DUX4* and *STAP1* expression levels in an independent BCP-ALL cohort (n = 2 *KMT2A*-rearranged, n = 1 *BCR-ABL1*, n = 21 B-other, n = 17 *ETV6-RUNX1*, n = 21 high hyperdiploid). Fastq-files with paired-end data were aligned to one DUX4 gene and the STAP1 GRCh37 reference sequence using STAR 2.5.0b. Read counts were determined with HTSeq-count version 0.6.1p1. Grey lines represent the median expression values. (**a**) *DUX4* expression values are depicted as Fragments Per Kilobase per Million mapped (FPKM). *DUX4*-partner genes were identified using NCBI blast, in which unknown sequences after the breakpoint were aligned^52^. *DUX4*-allignement of 4 *DUX4-*rearranged cases and 4 non*-DUX4*-rearranged B-other cases is shown. (**b**) *STAP1* expression values are depicted as FPKM.

Interestingly, *ERG* deletions were very recently shown to be a hallmark of a newly identified BCP-ALL subtype, involving rearrangements of the double homeobox transcription factor *DUX4*^8–10,31^. The association between high STAP1 levels and ERG deletions prompted us to investigate the presence of DUX4 rearrangements in our cases. To this aim, we screened an independent BCP-ALL cohort (n = 2 *KMT2A*-rearranged, n = 1 *BCR-ABL1*, n = 21 B-other, n = 17 *ETV6-RUNX1*, n = 21 high hyperdiploid) for *DUX4*-rearrangements and *ERG* deletions. Four *DUX4*-rearranged cases were detected in the B-other group, of which two showed high expression levels of *STAP1* (Fig. 7). None of these *DUX4*-rearranged cases or remaining B-other cases harbored an *ERG* deletion. In addition, we analyzed the association between STAP1, ERG, and DUX4 using a publically available dataset of 304 BCP-ALL cases^10,32^. Similar to the observation made in our own patients, *DUX4*-rearranged cases displayed higher levels of STAP1 mRNA (Supplementary Fig. S6), which was independent of intragenic *ERG* deletions.

Taken together, high expression of *STAP1* is not associated with (long-term) clinical outcome parameters or *ex vivo* drug resistance. However, the *STAP1*-high group is enriched for *DUX4*-rearrangements and intragenic *ERG* deletions.

## Discussion

The aim of the current study was to assess the potential of *STAP1* as a therapeutic target in BCP-ALL cases. High expression of *STAP1* was detected in a subset of *BCR-ABL1*-like and non-*BCR-ABL1*-like B-other patients. Interference with *STAP1* did not induce cell death or block proliferation nor did it make cells more sensitive to BTK (ibrutinib) and mTOR (rapamycin) inhibitors. In addition, high expression levels of *STAP1* were not associated with a poor five-year EFS, high MRD levels, or *ex vivo* resistance to chemotherapeutic drugs that are traditionally used in the treatment of BCP-ALL. Taken together, these results suggest that *STAP1* is not a likely target for precision medicines in childhood ALL.

*STAP1* is a relatively unknown gene. A potential role for the protein in neuronal apoptosis and degeneration has been suggested^26^. However, involvement of the protein in cancer thus far remains elusive. We studied the gene expression profiles of *STAP1*-high BCP-ALL cases. The observed association between high expression of STAP1 and BCR signaling is in concordance with the few reports suggesting that *STAP1* is a docking protein downstream of the BCR^24,25^. Targeting the BCR pathway is an effective treatment strategy for mature B-cell malignancies and this approach is also being explored in BCP-ALL^15–20^. The overlap in downstream molecules involved in pre-BCR and BCR signaling^14^, prompted us to investigate the connection between STAP1 expression and the pre-BCR pathway. However, CyIgµ expression (indicative for a functional pre-BCR) was not enriched in *STAP1*-high cases, and stimulation of leukemic cell lines with IgM only marginally induced expression *STAP1* in one cell line. In addition, silencing of *STAP1* did not reduce the proliferation rate and viability of leukemic cells in the majority of shRNAs tested. Strikingly, knockdown of *STAP1* did also not affect phosphorylation levels of marker proteins involved in pre-BCR signaling (SRC) or other signaling pathway (PI3K, Ras, Stat, JNK, p38, NFκB), which are important for proliferation and survival of cells. In addition, silencing of STAP1 did not affect the phosphorylated levels of CREB, which is a downstream target of STAP1 in the Ramos/Burkitt lymphoma cell line^25^. The question remains regarding the signaling pathway in which STAP1 is involved. Nevertheless, our results imply that *STAP1* is not an oncogenic driver and that the high expression is most likely a consequence of another transforming event.

Interestingly, we observed intragenic deletions of the transcription factor ERG in a part of the *STAP1*-high cases. The proto-oncogene *ERG* is a regulator of hematopoiesis, including B-cell development^33,34^, and is implicated in the pathogenesis of Ewing sarcoma, prostate cancer, and acute myeloid leukemia^35–37^. Recently, *ERG* deletions were shown to be a hallmark of a novel identified subtype of BCP-ALL, i.e. *DUX4*-rearranged ALL^8–10^. *DUX4* encodes a double homeobox transcription factor and is located within the D4Z4 macrosatellite repeat array on 4q35 and 10q26. This pro-apoptotic gene is normally expressed in germline and testis cells, but epigenetically silenced in somatic cells^38,39^. DUX4-fusion proteins, but not wildtype DUX4, were shown to have oncogenic potential in NIH3T3 fibroblasts^9^. The pathway induced by *DUX4*-rearrangements is yet unknown. In the present work we show that high expression of *STAP1* is characteristic for *DUX4*-rearranged cases (Fig. 7, Supplementary Fig. S6). The association between STAP1 and DUX4 was independent of *ERG* deletions. Therefore, upregulation of STAP1 may be a consequence of the oncogenic signaling pathway induced in *DUX4*-rearranged cases which is independent of concomitant *ERG* deletions. Further unraveling of the signaling pathway downstream of DUX4 will be crucial for the development of targeted treatment strategies for this genetic BCP-ALL subtype.

## Methods

A detailed description of all methods can be found in the supplement.

### Processing of primary patient material

Bone marrow and/or peripheral blood samples were obtained from children (1–18 years) with newly diagnosed ALL. Written informed consent was obtained from parents or guardians to use excess of diagnostic material for research purposes, as approved by the Medical Ethics Committee of the Erasmus Medical Center, The Netherlands. Studies were conducted in accordance with the Declaration of Helsinki. Mononuclear cells were isolated using Lymphoprep gradient separation and the leukemic blast percentage was determined microscopically by May-Grünwald Giemsa stained cytospin preparations, as described previously^40^. Samples were enriched to >90% leukemic cells by depleting normal cells, using anti-CD marker coated magnetic beads, i.e. anti-CD3, anti-CD14, anti-CD15, anti-CD33, and/or H1 beads (IQ Products, Groningen, The Netherlands) combined with pan mouse IgG dynabeads (Invitrogen, Bleiswijk, Netherlands). Primary leukemic cells were maintained in RPMI-1640 Dutch modification (Life Technologies, Breda, Netherlands) supplemented with 20% fetal calf serum (Integro, Zaandam, Netherlands), with 0.1% insulin-transferrin-sodium selenite (Sigma-Aldrich, Zwijndrecht, Netherlands), 0.4 mM glutamine (Invitrogen), 0.25 μg/ml gentamycine (Thermo Scientific, Breda, Netherlands), 100 IU/ml penicillin (Thermo Scientific), 100 μg/ml streptomycin (Thermo Scientific), 0.125 μg/ml fungizone (Thermo Scientific).

Patients were treated according to the Dutch Childhood Oncology Group ALL8, ALL9, ALL10 protocol, or the COALL-06-97 and COALL-07-03 study protocols^41–45^. The major subtypes (high hyperdiploid (51–65 chromosomes), *ETV6-RUNX1*, *TCF3-PBX1*, *KMT2A*-rearranged, *BCR-ABL1*) were determined using fluorescent *in situ* hybridization and (RT-)PCR by reference laboratories. Cases negative for these lesions were classified as B-other. Among these B-other cases, *BCR-ABL1*-like cases were identified using the 110-probeset gene expression classifier^2^. In addition, immunophenotyping was performed, including cytoplasmic Igμ (CyIgμ) expression. Samples containing more than 30% leukemic cells expressing CyIgμ were labelled as CyIgμ-positive.

### Cell lines

The human BCP-ALL cell lines Nalm6 and Kasumi-2 were obtained from the German Collection of Microorganisms and Cell lines (DSMZ, Braunschweig, Germany). Cells were cultured in RPMI-1640 medium, supplemented with 10% fetal calf serum (Bodinco BV, Alkmaar, Netherlands), 100 units/ml penicillin, 100 μg/ml streptomycin and 0.125 μg/mL fungizone (Life Technologies). The identity of cell line was routinely verified by DNA fingerprinting. The B-other cell line Nalm6 expressed high levels of *STAP1* and *TCF3-PBX1*-positive Kasumi-2 cells had low expression levels of *STAP1*. For stimulation experiments, cells were exposed to 1 µg anti-IgM F(ab′)2 (SouthernBiotech, Birmingham, AL, USA).

### *STAP1* expression status

Microarrays (Affymetrix U133 Plus 2 Santa Clara, California, USA) were analyzed of a previously published cohort of 572 BCP-ALL patients at initial diagnosis (GSE13351)^21^, in which all major ALL subtypes were represented (*BCR-ABL1*-positive n = 24, *BCR-ABL1*-like B-other n = 92, non-*BCR-ABL1*-like B-other n = 113, *ETV6-RUNX1*-positive n = 172, high hyperdiploid n = 141, *KMT2A*-rearranged n = 11, *TCF3-RUNX1*-positive n = 19). Gene expression profiles of *BCR-ABL1*-like and non-*BCR-ABL1*-like B-other samples were compared to remaining BCP-ALL cases, using Limma R Package (version 3.26.9) in R 3.0.1. *BCR-ABL1*-like and non-*BCR-ABL1*-like B-other cases with signal intensity values of both STAP1 probesets (220059_at and 1554343_a_at) above the 80^th^ percentile of *BCR-ABL1*-like and non-*BCR-ABL1*-like B-other cases were classified as *STAP1*-high (see also Fig. 1a). Remaining *BCR-ABL1*-like and non-*BCR-ABL1*-like B-other cases were classified as *STAP1*-low.

### Quantitative reverse transcription PCR (RT-qPCR)

mRNA expression levels of *STAP1* and *RPS20* were quantified using real-time PCR analysis on an ABI Prism 7700 sequence detection system (PE Applied Biosystems). RNA was extracted using the RNeasy mini kit (Qiagen, Hilden, Germany), after which cDNA was synthesized. *STAP1* and *RPS20* mRNA levels were quantified by incorporation of SYBR Green (Thermo Scientific) by quantitative real-time PCR, using *STAP1*-specific primers (5′-ccaggaaaggttaaagattact-3′ and 5′-ttccccactttctgtgtt-3′) and *RPS20*-specific primers (5′-aagggctgaggatttttg-3′ and 5′-cgttgcggcttgttag-3′). Relative *STAP1* mRNA levels as percentage of RPS20 levels were calculated using the comparative cycle time (Ct) method; 2^−ΔCt^ × 100%, whereby ΔCt = Ct_*STAP1*_ − CT_*RPS20*_.

### Transfection, virus production and transduction

To knockdown *STAP1* expression, four pLKO.1-puro Mission® vectors (Sigma-Aldrich) containing a short hairpin RNA (shRNA) targeting *STAP1* (TRCN0000065083, TRCN0000065084, TRCN0000065085, TRCN0000065087; shRNA-1, shRNA-2, shRNA-3, shRNA-4, respectively) were used. The Mission® pLKO.1-puro Non-Mammalian shRNA Control Plasmid DNA (SHC002) and Mission^®^ pLKO.1-puro Luciferase shRNA Control Plasmid DNA (SHC007) were used as scrambled control vectors (NSC-1 and NSC-2, respectively).

Vectors were transfected in HEK293T cells, using XtremeGene, 3.7 μg psPAX2 (Addgene plasmid 12260; Addgene, Cambridge, MA, USA), 1.6 μg pMD2.G (Addgene plasmid 12259) and 10.7 μg of one of the pLKO.1-puro Mission® vectors. The second and third day after transfection virus was harvested and concentrated using ultracentrifugation for 2 hours at 32.000 rpm and 4 °C. Concentrated virus was aliquoted and stored at −80 °C. Leukemic cells were spin-infected and puromycin selection was initiated 24 hours after transduction. Transduction efficiency was determined using a titration range. After 48 hours of puromycin selection (1 µg/ml) cell viability was measured using flow cytometry (MACSQuant) and propidium iodide (PI; Invitrogen). Viability of transduced cells as percentage of viability of non-transduced cells was defined as the transduction efficacy. Proliferation was measured for 5 days using flow cytometry (MACSQuant) and PI staining. At day 5, viability was quantified using Annexin V (Biolegend, London, UK) and PI staining.

### *Ex vivo* drug resistance

*Ex vivo* cytotoxicity of prednisolone, vincristine, L-asparaginase, daunorubicin, 6-mercaptopurine and 6-thiguanine in primary samples was evaluated using 3-(4,5-dimethylthiazolyl-2)-2,5-diphenyltetrazolium bromide (MTT), as previously described^40^. After four days of culture, viability was quantified by measuring the optical density values after 6 hours of incubation with MTT. LC50 values were compared using the Mann-Whitney U test. *In vitro* cytotoxicity of prednisolone, ibrutinib and rapamycin in cell lines was evaluated using 3-(4,5-dimethylthiazolyl-2)-5-(3-carboxymethoxyphenyl)-2-(4-sulfophenyl)-2H-tetrazolium; MTS) and phenazine methosulfate (PMS). Cells were exposed to a dilution series of agents (prednisolone: 0.06 to 250 μg/mL; ibrutinib: 0.16 to 40 μM; rapamycin: 4 nM to 40 μM) in a 96 wells plate for four days at 37 °C and 5% CO_2_, after which viability was quantified.

### Multiplexed fluorescent bead-based immunoassay (Luminex)

Cells were lysed in RIPA buffer (ThermoFisher Scientific) with freshly added protease and phosphatase inhibitors. Protein concentration was determined using the BCA method (Thermo Scientific). Expression of 17 proteins was determined in 10 µg lysate, using a fluorescent bead-based immunoassay (multi-pathway magnetic bead 9-plex and the 8-plex human Src family kinase kit; Merck Millipore, Amsterdam, Netherlands) according to the manufacturer’s protocol. GAPDH beads (Merck Millipore) were used as internal reference for each sample.

### Multiplex Ligation-Dependent Probe Amplification (MLPA)

To identify genomic lesions in *IKZF1*, *CDKN2A*, *CDKN2B*, *ETV6*, *PAX5*, *RB1*, *BTG1*, *EBF1*, and *PAR1* (*CSF2RA/IL3RA/CRLF2*), the SALSA P335 ALL-*IKZF1* (a3) and the SALSA P202 Multiplex Ligation-dependent Probe Amplification (MLPA) assays (MRC-Holland, Amsterdam, Netherlands) were used as described previously^4,21,46^. A peak ratio < 0.75 was used to determine deletions, 0.75 ≤ peak ratio ≤ 1.3 for normal copy number, and peak ratio > 1.3 for gain. Loss of either *CDKN2A* or *CDKN2B* was coded as *CDKN2A/B* deletion and intragenic amplifications of *PAX5* were coded as aberration.

### Genome-wide DNA copy number arrays (array-CGH)

To identify *ERG* deletions, genome-wide DNA copy number arrays were performed as described previously^4^. Briefly, Agilent SurePrint G3 Hmn 4 × 180 K arrays (Agilent Technologies, Amstelveen, the Netherlands) were co-hybridized with 1 μg patient DNA labeled with ULS-Cy5 and 1 μg reference genomic DNA male pool (G147A, Promega, Leiden, the Netherlands) labeled with ULS-Cy3 (Agilent Genomic DNA ULS Labeling Kit). Using median log ratios, data were normalized using the CGHcall^47^ version 2.14.0, centralized using CGHnormaliter^48^ version 1.8.0, and segmented and called using CGHcall default settings (−1 for loss, 0 for diploid, 1 for gain and 2 for amplification) in R version 2.14.1.

### Clinical characteristics and statistics

To identify whether copy number alterations (CNAs), clinical characteristics or CyIgμ expression were depleted or enriched in cases harboring high expression levels of *STAP1*, the Fisher’s exact test in R (version 3.2.1) was applied. Cumulative incidence of relapse (CIR) was estimated using a competing risk model and significance was determined using the Gray’s test. Relapse and non-response (counted at day 79 of therapy) were considered as events and death as competing event. Event-free survival (EFS) probabilities were estimated using cox regression and compared using the Wald test. Relapse, non-response, secondary malignancies and death were counted as events. Outcome analyses were performed in R (version 3.2.1), using the packages cmprsk version 2.2-7^49^, mstate version 0.2.7^50^, and survival version 2.37-4^51^. Five-year EFS and CIR are reported.

### SNP arrays

Genome-wide human SNP arrays 6.0 (Affymetrix) were performed according to the manufacturer’s protocol. Raw probe values were extracted from CEL files and processed with the R package aroma.affymetrix version 3.1.0. Samples were compared to reference values, which was the average of 53 diploid BCP-ALL and T-ALL samples. To correct for bias introduced by differences in GC content of DNA fragments, the R package ArrayTV version 1.12.0 was used. To compare the logR values of the copy numbers between samples a centralization step was performed, using the R package CGHnormaliter version 1.28.0. Data were called using CGHcall version 2.36.0 default settings (−2 for double loss, −1 for loss, 0 for diploid, 1 for gain and 2 for amplification) in R version 3.3.3.

### RNAseq

mRNA was extracted from total RNA and amplified using random hexamer primers. Further Library construction was done using a strand-specific protocol. Sequencing was performed on a HiSeq. 2000 producing 151 bp paired-end reads with a median library size of 50 million read pairs per sample. Fastq-files with paired-end data were aligned to the GRCh37 reference sequence using STAR 2.5.0b. Read counts were determined with HTSeq-count version 0.6.1p1. To determine *DUX4* expression, the fastq-files were aligned to the DNA sequence of the gene ENSG00000259128.1 plus 200 bp down- and upstream. *DUX4*-partner genes were identified using NCBI blast, in which unknown sequences after the breakpoint were aligned^52^.

### Data availability

The datasets generated during and/or analyzed during the current study are available from the corresponding author on reasonable request.

## Electronic supplementary material

Supplementary information

Supplementary Table 1-3
