# Supplementary material for: High STAP1 expression in DUX4-rearranged cases is not suitable as therapeutic target in pediatric B-cell precursor acute lymphoblastic leukemia
Source: Sci Rep. 2018 Jan 12;8:693. doi: 10.1038/s41598-017-17704-4 (PMC5766593; doi:10.1038/s41598-017-17704-4)

High STAP1 expression in DUX4-rearranged cases is not suitable as therapeutic target in pediatric B-cell precursor acute lymphoblastic leukemia

Elisabeth M.P. Steeghs[1], Marjolein Bakker[1], Alex Q. Hoogkamer[1], Judith M. Boer[1,2], Quirine J. Hartman[1], Femke Stalpers[1], Gabriele Escherich[3], Valerie de Haas[4], Hester A. de Groot-Kruseman[4], Rob Pieters[2,4], and Monique L. den Boer[1,2,4]

[1] Department of Pediatric Oncology/Hematology, Erasmus Medical Center – Sophia Children's Hospital, Rotterdam, the Netherlands

[2] Princess Máxima Center for Pediatric Oncology, Utrecht, The Netherlands.

[3] COALL - German Cooperative Study Group for Childhood Acute Lymphoblastic Leukemia, University Medical Centre Eppendorf, Martinistrasse 52, 20246 Hamburg, Germany

[4] DCOG, Dutch Childhood Oncology Group, The Hague, The Netherlands

Correspondence:

Monique L. den Boer, PhD m.l.denboer@erasmusmc.nl

Erasmus MC - Sophia Children's Hospital

Department of Pediatric Oncology/Hematology, room Sp-2435

Wytemaweg 80

NL-3015 CN Rotterdam, The Netherlands

Phone: +31 107043936 or +31 107036691

Fax: +31 107044761

Supplementary methods

Microarray data analysis

Gene expression profiles of *BCR-ABL1*-like and non-*BCR-ABL1*-like B-other *STAP1*-high and *STAP1*-low samples were compared, using Limma R Package (version 3.26.9) in R 3.0.1. To identify canonical pathways that were more significantly different, ingenuity pathway analysis software was used (QIAGEN Inc., https://www.qiagenbioinformatics.com/products/ingenuitypathway-analysis).

Western Blotting

Leukemic cells were lysed in lysis buffer supplemented with freshly added protease and phosphatase inhibitors. 25μg (BCA method; Thermo Scientific) lysate was loaded on 10% mini protean precast gels (BioRad, Veenendaal, Netherlands), and transferred to a nitrocellulose membrane (Biorad). Primary antibody incubation was performed according to manufacturer's protocol. Anti-pAKT^{Ser473} and anti-αTubulin (#2144) were obtained from Cell Signaling Technology (Danvers, Massachusetts, USA). Anti-STAP1 (H00026228-M01) was obtained from Abnova (Aachen, Germany) and anti-β-actin (ab6276) was obtained from Abcam (Cambridge, UK). Blots were stained with secondary antibodies (IRDye 680RD- or 800CW-labelled anti-rabbit and IRDye 680RD- or 800CW-labelled anti-mouse; Li-Cor Biosciences, Leusden, Netherlands) and scanned using the Odyssey infrared imaging system (Li-Cor Biosciences). Expression levels were quantified using Odyssey application software version 3.0.21.

***Ex vivo* drug resistance**

Ex vivo cytotoxicity of prednisolone, vincristine, L-asparaginase, daunorubicin, 6-mercaptopurine and 6-thiguanine in primary samples was evaluated using 3-(4,5-dimethylthiazolyl-2)-2,5-diphenyltetrazolium bromide (MTT). Cells were exposed to increasing concentrations of chemotherapeutic agents (prednisolone: 0.008 to 250 μg/mL; vincristine: 0.05 to 50 μg/mL; L-asparaginase: 0.003 to 10 IU/mL; daunorubicin: 0.002 to 2 μg/ml, 6-mercaptopurine: 0.015625 to 0.5 μg/ml, and 6-thioguanine: 0.001625 to 0.0.05 μg/ml) at 37°C and 5% CO_2. After four days of culture, the percentage of leukemic cells in control wells without chemotherapeutic agents was determined. Moreover, the optical density values of all wells was measured using a spectrophotometer after 6 hours of incubation with MTT. Samples were included if control wells harbored more than 70% leukemic cells and an optical density higher than 0.050

arbitrary units (adjusted for blank values). The concentration of drug lethal to 50% of the cells (LC50) was calculated. LC50 values were compared using the Mann-Whitney U test. *In vitro* cytotoxicity of prednisolone, ibrutinib and rapamycin in cell lines was evaluated using 3-(4,5-dimethylthiazolyl-2)-5-(3-carboxymethoxyphenyl)-2-(4-sulfophenyl)-2H-tetrazolium; MTS) and phenazine methosulfate (PMS). Cells were exposed to a dilution series of agents (prednisolone: 0.06 to 250 µg/mL; ibrutinib: 0.16 to 40 µM; rapamycin: 4 nM to 40 µM) in a 96 wells plate for four days at 37°C and 5% CO_2, after which viability was quantified as described above.

Multiplex Ligation-Dependent Probe Amplification (MLPA)

To identify genomic lesions in *IKZF1*, *CDKN2A*, *CDKN2B*, *ETV6*, *PAX5*, *RB1*, *BTG1, EBF1*, and *PAR1* (*CSF2RA/IL3RA/CRLF2*), the SALSA P335 ALL-*IKZF1* (a3) and the SALSA P202 Multiplex Ligation-dependent Probe Amplification (MLPA) assays (MRC-Holland, Amsterdam, Netherlands) were used as described previously.[1-3] Briefly, to generate DNA fragments with incorporated FAM nucleotides, 125 ng gDNA was used according to the manufacturer's protocol. Amplified fragments were quantified using an ABI-3130 genetic analyzer (Applied Biosystems, Carlsbad, CA). Manufacturer's control probes and a synthetic control reference, generated from five normal DNA samples in the same MLPA run, were used to normalize peak intensities (peak ratio < 0.75 for deletions, 0.75 ≤ peak ratio ≤ 1.3 for normal copy number, peak ratio > 1.3 for gain). Loss of either *CDKN2A* or *CDKN2B* was coded as *CDKN2A/B* deletion and intragenic amplifications of *PAX5* were coded as deletions.

SNP arrays

Genome-wide human SNP arrays 6.0 (Affymetrix) were performed to determine copy number and SNP genotype status according to the manufacturer's protocol. Raw probe values were extracted from CEL files and processed with the R package aroma.affymetrix version 3.1.0. This package contains a method to prepare the raw data for analysis using the CRMA v2 method. Arrays were processed individually to reduce the effects of probe crosstalk, nucleotide-position probe sequence and fragment-length. In addition, probe summarization, including copy number values from the SNP probesets, were acquired. Samples were compared to a reference values, which was the average of 53 diploid BCP-ALL and T-ALL samples. Copy number arrays can contain a very large bias between probes caused by differences in GC content of DNA fragments. This bias is likely introduced by the many amplification steps used in

the protocols for the arrays. We corrected for this bias using the R package ArrayTV version 1.12.0. To compare the logR values of the copy numbers between samples a centralization step was performed, using the R package CGHnormaliter version 1.28.0. Data were called using CGHcall version 2.36.0 default settings (-2 for double loss, -1 for loss, 0 for diploid, 1 for gain and 2 for amplification) in R version 3.3.3.

To determine the specificity and sensitivity of these SNP arrays, data were compared to array-CGH and MLPA data. The methods is excellent to call large copy number changes (larger than 1Kb, if many probes are present). However, it is difficult to see deletion of a single exon even in a large exon.

Supplementary Figure Legends

Supplementary Figure 1. Discriminative expression of *STAP1* in *BCR-ABL1*-like and B-other cases

(**A-B**) ^{2}log expression levels of Affymetrix probeset 220059_at in 572 BCP-ALL and 80 T-cell ALL (T-ALL) cases. The dotted line represents the 80th percentile of *BCR-ABL1*-like and B-other cases (**A**). Expression levels of nBM (n=6) samples were determined in another microarray batch, in which also T-ALL samples (n=105) were present (**B**). Grey lines represent the median expression values.

(**C-E**) ^{2}log expression levels of Affymetrix probeset 1554343_a_at in 654 BCP-ALL cases. The dotted line represents the 80th percentile of *BCR-ABL1*-like and B-other cases (**C**). Expression levels of nBM samples were determined in another microarray batch, in which also T-ALL samples were present (**D**). Grey lines represent the median expression values. Microarray expression levels were validated using qRT-PCR. Correlation between microarray values of probeset 1554343_a_at and qRT-PCR values is depicted by the spearman coefficient of correlation (**E**).

(**F**) Ingenuity pathway analysis software (Qiagen) was used to identify differentially regulated pathways between *STAP1*-high and *STAP1*-low *BCR-ABL1*-like and B-other cases was performed. Limma was applied to unravel different expressed probeset. Adjusted p-values below 0.05 were used to perform analysis.

Supplementary Figure 2. Stimulation of pre-BCR signaling

Nalm6 and Kasumi-2 cells were stimulated for 1 hour with 1 µg/ml anti-IgM. Phosphorylation levels of $pAKT^{Ser473}$ were detected using western blotting, to confirm the stimulatory effect of the anti-IgM antibody. Expression levels relative to β-actin are depicted.

Supplementary Figure 3. Virus titration to determine the amount of virus required to infect 80% of cells.

Nalm6 and Kasumi-2 cells were exposed to indicated dilution series of virus. Cells were transduced via spin-infection. Puromycin selection (1 µg/ml) was initiated 24 hours after infection. After 48 hours of selection, cell viability was assessed using flow cytometry. PI staining was used to discriminate viable from dead cells. Transduction efficacy was calculated: (%viability of transduced cells)/(%viability of non-

transduced cells)*100%. Obtained values of four independent experiments are depicted in graphs. The virus amounts used for subsequent experiments are indicated by enlarged symbols.

Supplementary Figure 4. Silencing of *STAP1* in BCP-ALL cell lines.

Nalm6 and Kasumi-2 cells were transduced via spin-infection with shRNAs targeting *STAP1* or scrambled control vectors.

(**A**) Target sequences and regions of the shRNAs are depicted in the Table.

(**B**) Western blot analysis of 15µg lysate of Nalm6 samples. Cell lysate was collected 3 days after transduction. The blot was probed with anti-STAP1 (mouse) and anti-αTubulin (rabbit; same blot). Protein expression was visualized using secondary antibodies (IRDye 680RD-labelled anti-rabbit and IRDye 800CW-labelled anti-mouse) and scanned using the Odyssey infrared imaging system. The cropped blot is shown and the full length blot is depicted in panel d. Quantified expression levels are shown above each band.

(**C**) Knockdown efficacy of the different shRNAs determined on protein level (western blot) and mRNA level (RT-qPCR), 3 days after transduction. Expression levels of STAP1 were normalized towards house-keeping genes (α-Tubulin and RPS20). Relative expression values towards the two scrambled controls are depicted.

(**D**) Full western blot image of cropped image that are depicted in panel b.

Supplementary Figure 5. Fluorescent bead-based immunoassay.

Expression of 17 proteins was determined using a fluorescent bead-based immunoassay in Nalm6 cells after knockdown of *STAP1*. Fluorescent intensity values relative to GAPDH are depicted. Expression was measured three and seven days after transduction. At day 7, not enough material was available of Nalm6 cells, which were transduced with shRNA-2. Values represent mean±SEM of four independent experiments at three days after transduction and three independent experiments at seven days after transduction. In addition, positive and negative cell line samples were included to validate the assay and antibodies. For the 8-plex human Src family kinase kit control samples included Hela-unstimulated lysate, Ramos-Pervanadate lysate, HL60- Pervanadate lysate, and HEK293 serum. For the multi-pathway magnetic bead 9-plex control samples included HeLa-unstimulated lysate, HeLa-TNFα/Calyculin lysate, A431-EGF lysate, MCF7-IGF1 lysate.

Supplementary Figure 6. *STAP1* expression and *DUX4*-rearrangemets.

STAP1 expression levels in 304 BCP-ALL cases, obtained ProteinPaint.[4,5] Fragments Per Kilobase per Million mapped (FPKM) expression values are depicted on the y-axis. Grey lines represent the median expression values. NOS = not other specified

Literature

1 van der Veer, A. *et al.* Independent prognostic value of BCR-ABL1-like signature and IKZF1 deletion, but not high CRLF2 expression, in children with B-cell precursor ALL. *Blood* **122**, 2622-2629, doi:blood-2012-10-462358 [pii] 10.1182/blood-2012-10-462358 (2013).

2 Boer, J. M. *et al.* Tyrosine kinase fusion genes in pediatric BCR-ABL1-like acute lymphoblastic leukemia. *Oncotarget* **8**, 4618-4628, doi:10.18632/oncotarget.13492 (2017).

3 Boer, J. M. *et al.* Expression profiling of adult acute lymphoblastic leukemia identifies a BCR-ABL1-like subgroup characterized by high non-response and relapse rates. *Haematologica* **100**, e261-264, doi:10.3324/haematol.2014.117424 (2015).

4 Zhou, X. *et al.* Exploring genomic alteration in pediatric cancer using ProteinPaint. *Nat Genet* **48**, 4-6, doi:10.1038/ng.3466 (2016).

5 Zhang, J. *et al.* Deregulation of DUX4 and ERG in acute lymphoblastic leukemia. *Nat Genet* **48**, 1481-1489, doi:10.1038/ng.3691 (2016).

Supplementary Figure 1. Discriminative expression of *STAP1* in *BCR-ABL1*-like and B-other cases

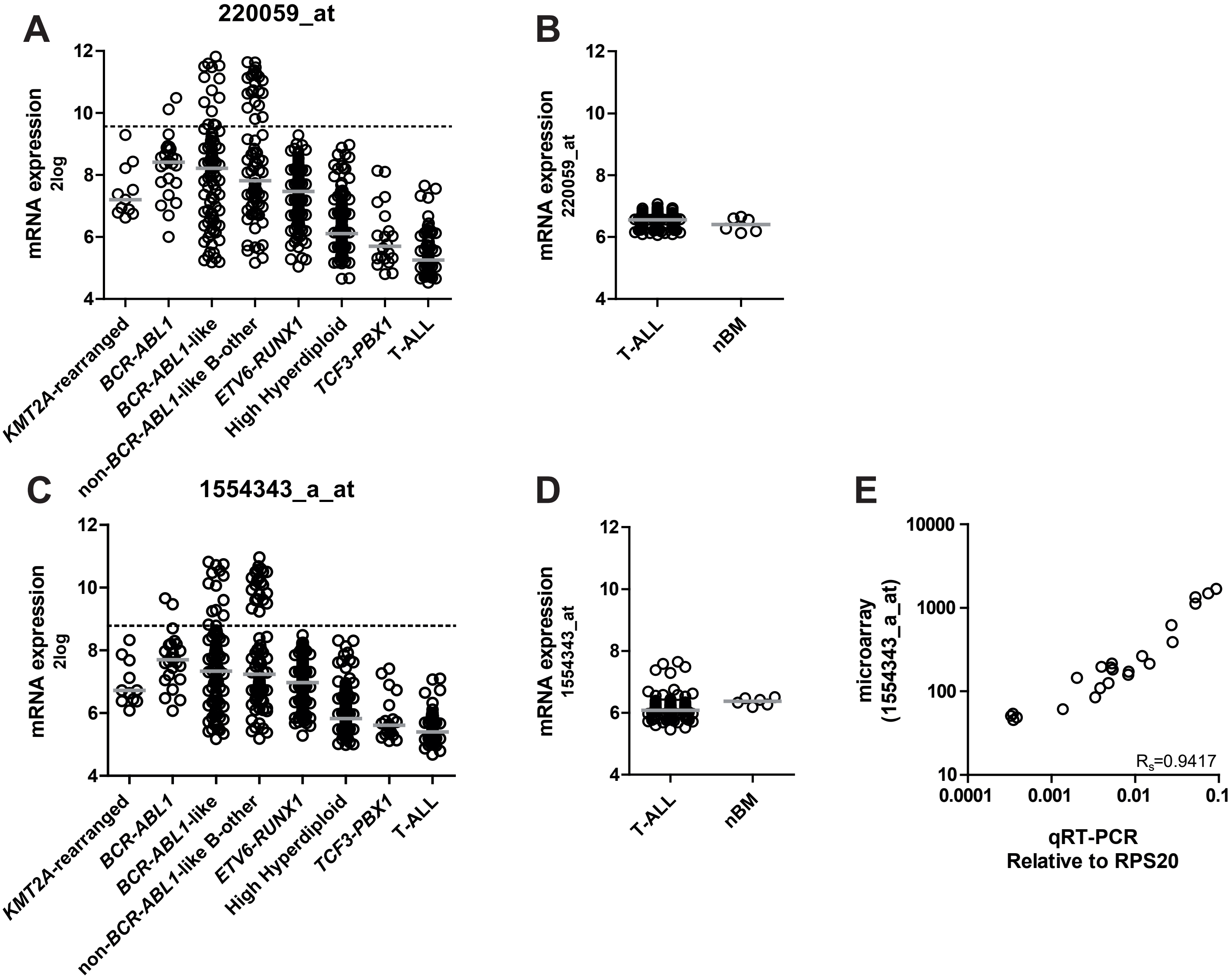

F

Ingenuity canonical pathways	Overlapping genes	P-value
EIF2 Signaling	66.5% (129/194)	2.18E-21
mTOR Signaling	58.8% (117/199)	1.08E-13
Regulation of eIF4 and p70S6K Signaling	59.9% (94/157)	6.50E-12
B Cell Receptor Signaling	54.6% (101/185)	1.68E-09
AMPK Signaling	52.4% (99/189)	4.10E-08

Supplementary Figure 2. Stimulation of pre-BCR signaling

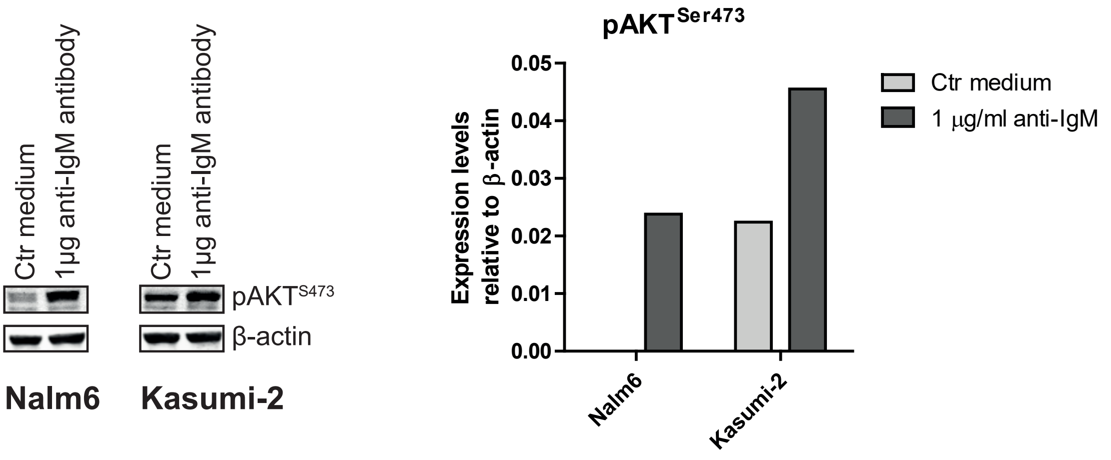

Supplementary Figure 3. Virus titration to determine the amount of virus required to infect 80% of cells.

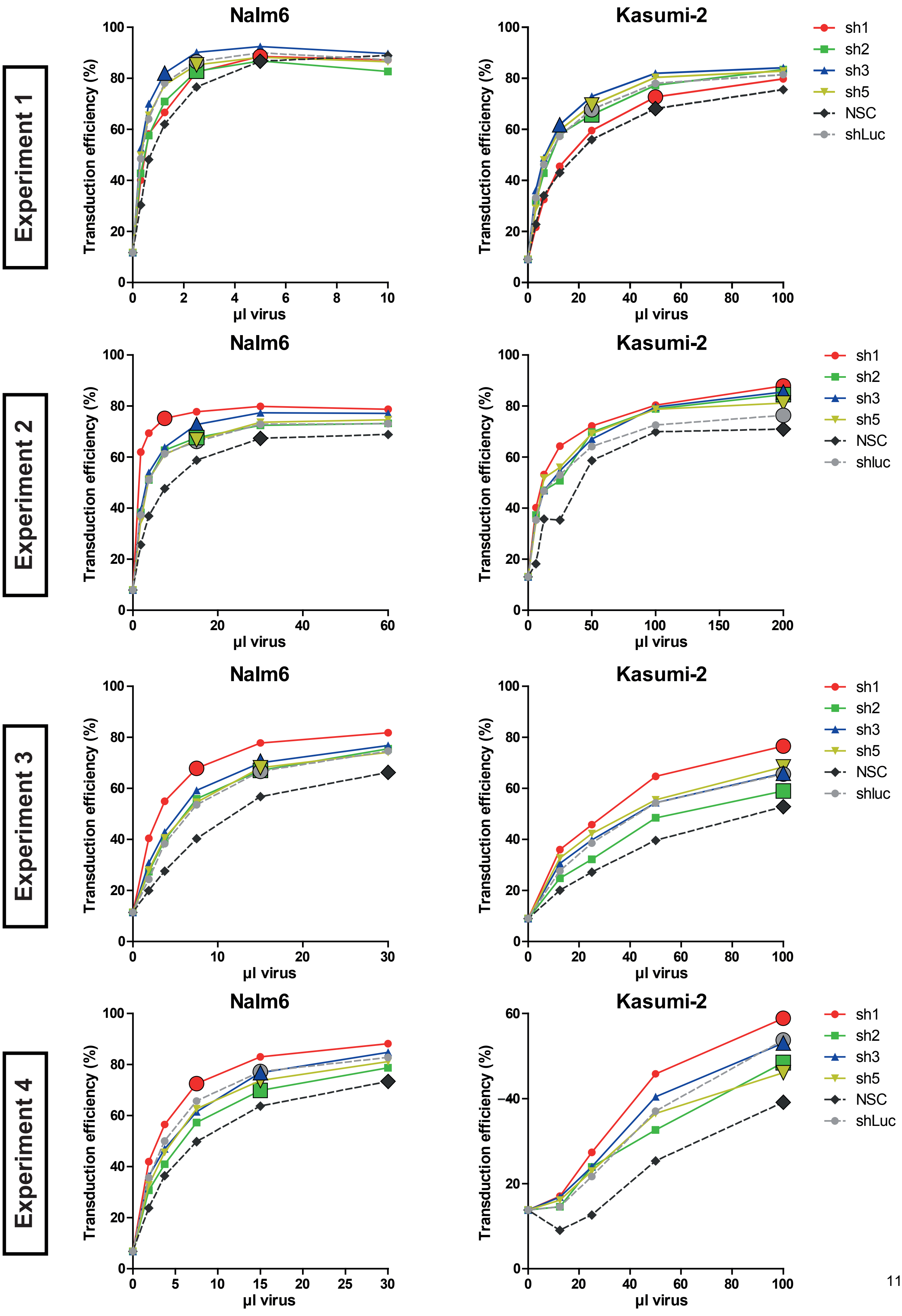

Supplementary Figure 4. Silencing of *STAP1* in BCP-ALL cell lines.

a

	Clone ID	Target Sequence	Target region
**shRNA-1**	TRCN0000065083	CCAAGAATCAAGCACTACAAA	Exon 7
**shRNA-2**	TRCN0000065084	GCTCTACCTTTGTACTTTGAA	Exon 1
**shRNA-3**	TRCN0000065085	GCCTGGTAGTGACAGTAGAAA	Exon 6
**shRNA-4**	TRCN0000065087	CCTGTAACACTCCCAAACCTT	Exon 7/8

b

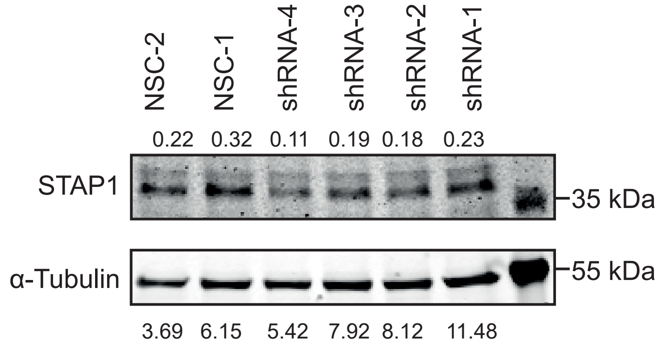

c

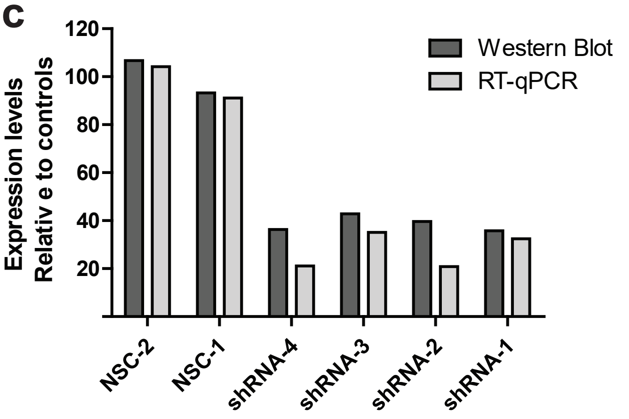

d

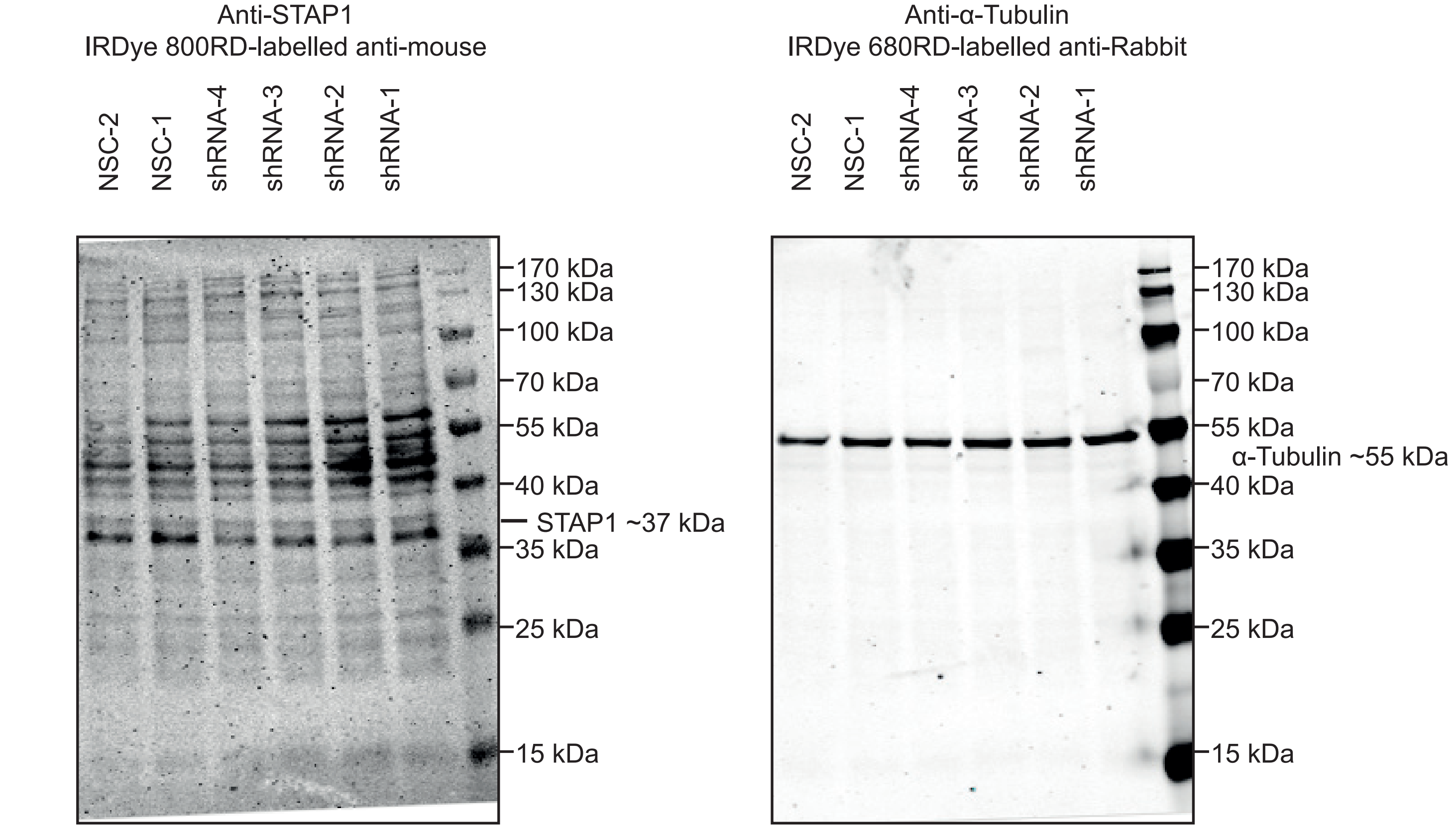

Supplementary Figure 5. Fluorescent bead-based immunoassay

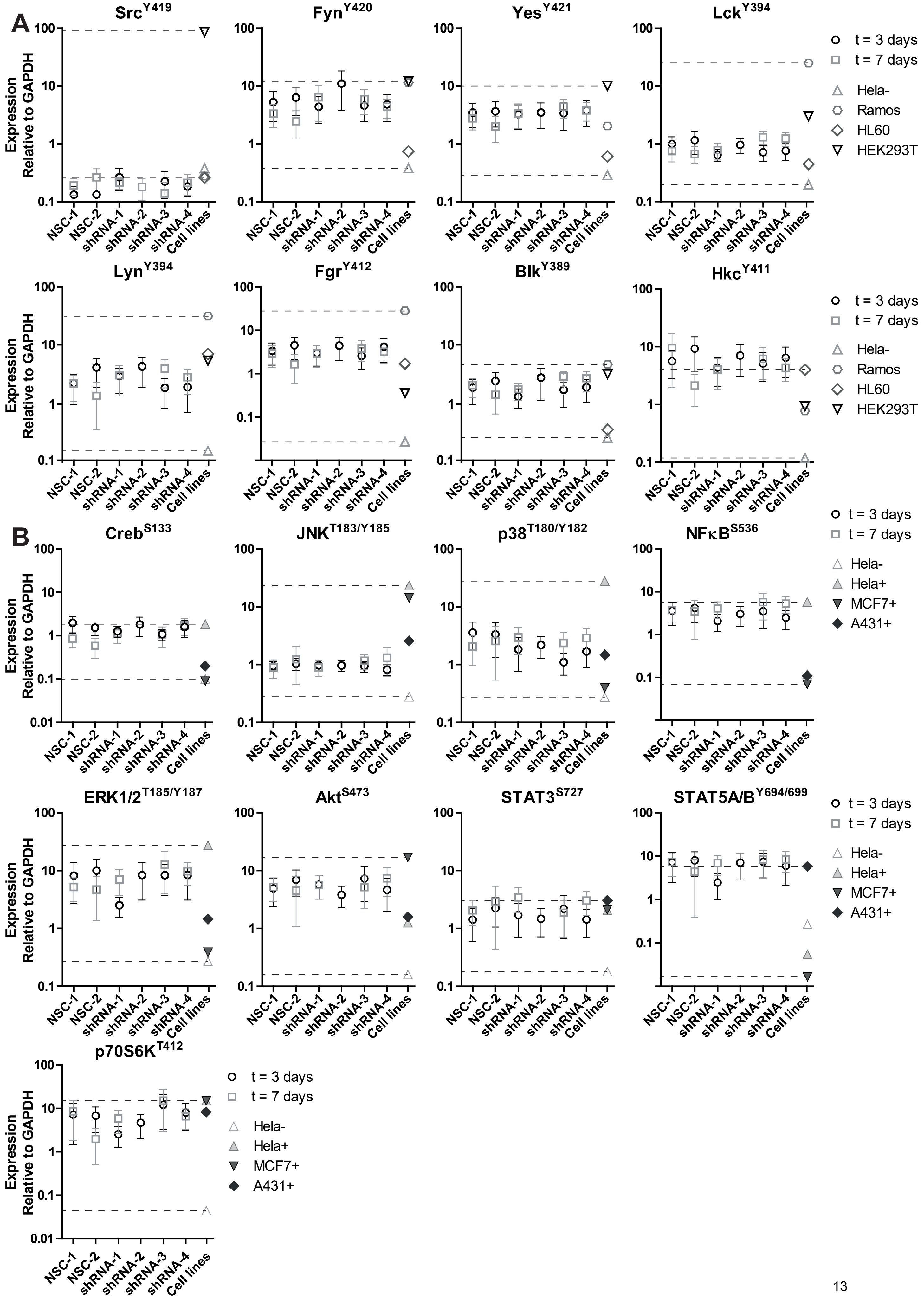

Supplementary Figure 6. *STAP1* expression and *DUX4*-rearrangements

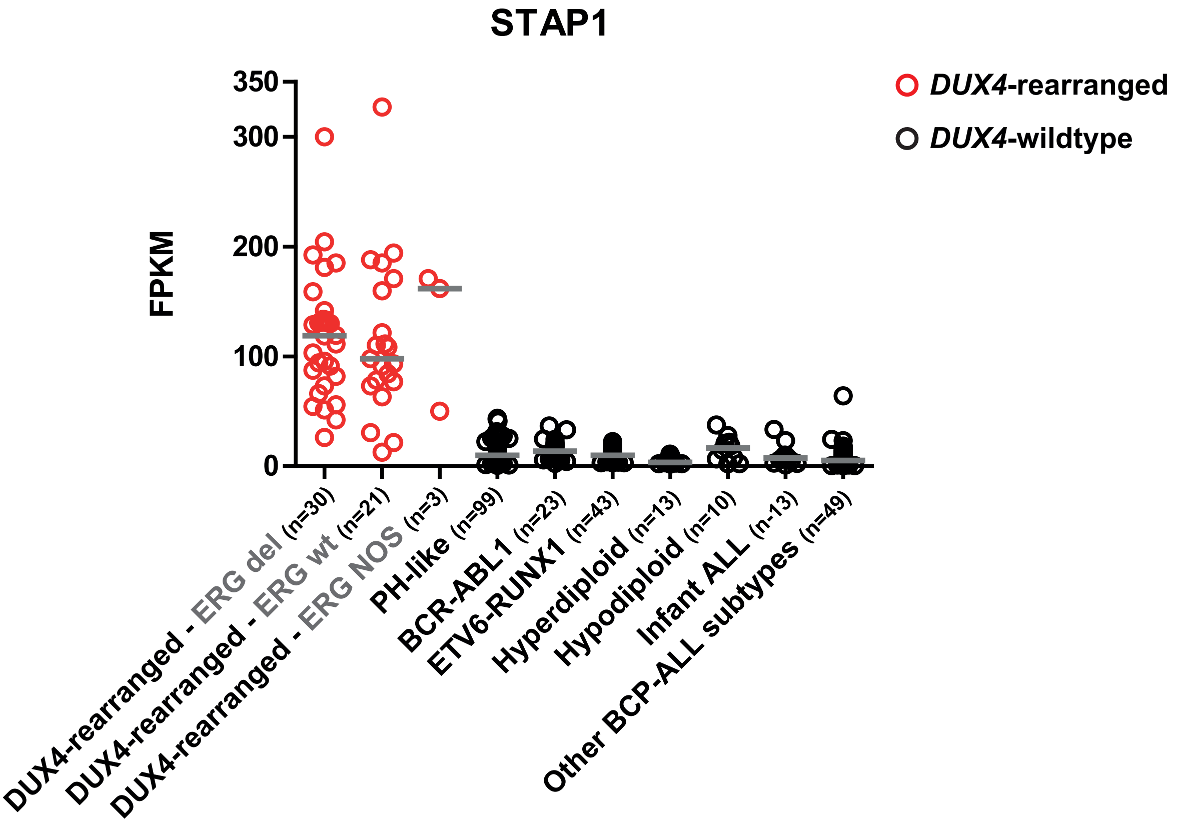

Supplement: Supplementary file 1 — Supplementary information [file 41598_2017_17704_MOESM1_ESM.pdf]
